# (*Z*)-1,2-Bis(4-nitro­phen­yl)ethene. Corrigendum

**DOI:** 10.1107/S1600536808009999

**Published:** 2008-04-16

**Authors:** Guanfan Chen, Chenzhong Cao

**Affiliations:** a School of Chemistry and Chemical Engineering, Central South University, Changsha, Hunan 410083, People’s Republic of China; b School of Chemistry and Chemical Engineering, Hunan University of Science and Technology, Xiangtan, Hunan 411201, People’s Republic of China

## Abstract

The name of the first author in the paper by Chen & Cao [*Acta Cryst.* (2007), E**63**, o3999] is corrected.

## Abstract 2

Corrigendum to *Acta Cryst.* (2007), E**63**, o3999.

In the paper by Chen & Cao [*Acta Cryst.* (2007), E**63**, o3999], the name of the first author is incorrect. The correct name is given above.

